# Spatial patterns of genetic diversity, community composition and occurrence of native and non-native amphipods in naturally replicated tributary streams

**DOI:** 10.1186/s12898-016-0079-7

**Published:** 2016-04-26

**Authors:** Florian Altermatt, Roman Alther, Elvira Mächler

**Affiliations:** Department of Aquatic Ecology, Eawag, Swiss Federal Institute of Aquatic Science and Technology, Überlandstrasse 133, 8600 Dübendorf, Switzerland; Department of Evolutionary Biology and Environmental Studies, University of Zurich, Winterthurerstr. 190, 8057 Zurich, Switzerland

**Keywords:** Invasive species, Invasion process, Dispersal, *Gammarus*, *Dikerogammarus*, Stream network, Lake Constance, Meta-community, Microsatellites

## Abstract

**Background:**

Worldwide, natural communities are invaded by non-native species, with potentially devastating effects on the native communities. A large part of past research aimed at finding traits and characteristics of the invading species or the invaded community explaining observed invasions. Only recently, the focus shifted on the spatial patterns during invasions per se. Empirical data, however, are limited, as invasions are often unique incidences of a complex spatio-temporal process. In order to identify generalities of invasion patterns, we studied 13 naturally replicated tributary streams draining into Lake Constance, and studied the occurrence of native and non-native amphipods along linear transects from the stream outlets to the upstream headwater reaches.

**Results:**

We found repeated spatial patterns of community composition and the occurrence of native and non-native amphipod species across two different years. Specifically, occurrence as well as abundance of two non-native amphipod species decreased from the stream outlets at the lake site towards upstream headwater reaches. Populations of the most common native amphipod species were largest at the uppermost headwater reaches. All populations of this native species, however, showed significant signals of recent genetic bottlenecks, irrespective of the stream position and occurrence of non-native species. Contrary to our expectations, this native species also showed no longitudinal genetic differentiation within individual tributaries as postulated for headwater versus outlet populations.

**Conclusions:**

Our results indicate that invasions of river-systems may overall follow predictable patterns on the level of spatial distributions and community composition. However, effects of invading organisms on the genetic diversity and genetic structure of native populations observed at larger scales may not necessarily be directly reflected at the scale of smaller tributaries.

**Electronic supplementary material:**

The online version of this article (doi:10.1186/s12898-016-0079-7) contains supplementary material, which is available to authorized users.

## Background

Natural communities are invaded by non-native species at a global scale. Many of these non-native species have large effects on natural communities and are, next to climate and habitat change, seen as the strongest driver of global biodiversity changes [1]. Thus, there is a high need to understand the spread of non-native species and subsequent effects on the composition of native communities as well as the genetic diversity and structure of native populations. Much past research on non-native species has either been on finding specific traits linked to invasion success of non-native species, or on characteristics making local communities/habitats more prone to invasions (e.g., [2–5]). Spatial patterns linked to the invasion process itself received less attention, even though the spread process is both typical for invasions (e.g., [6–8]) and relevant in the general context of dispersal and range expansions [9–11].

Early work, for example by Skellam [12], found invasions to be highly predictable by species-specific growth rates and diffusion coefficients, and the validity of this process has recently been experimentally confirmed [13]. Invasions of invertebrates, such as amphipods, could be described in similar ways, for example in the well-documented invasion of the river Rhine [7], where the invading species completely shifted abundance and structure of native invertebrate species communities. However, in many real-world cases, each invasion into a specific ecosystem is a unique incidence without spatial or temporal replication. Indeed, conservation measures seek to prevent invasions from repeating and ethical principles discourage experimental replication of invasions [6]. Thus, empirical data on the spatio-temporal unfolding of invasions are rare [14] or are confined to experimental studies in laboratory systems (e.g., [15]) or to examples of globally invasive species that have been introduced manifold (e.g., [14, 16]). The lack of naturally replicated invasions also at smaller scales is reducing our ability to derive general principles regarding invasion patterns, and especially reduces our ability to better understand the variance of invasion processes. This is especially unfortunate in the context of a continuing worldwide proliferation of invasions.

A possible path to a better understanding of invasion processes and subsequent effects on natural communities is to study naturally replicated systems exhibiting similar spatial and environmental structures, and potentially experiencing similar invasion pressure. A prime example of naturally replicated systems are individual tributaries within river networks [17, 18]. Individual tributaries reflect spatially independent habitat networks; species are often introduced at harbours or large downstream rivers, and invasion processes subsequently occur in the upstream direction (e.g., [7]). Thereby, each individual tributary stream may offer a unique incidence of invasion and the hierarchical and fractal structure of river systems may allow us to examine composition of the natural communities and occurrence of invading species in a spatially “replicated” setting. Understanding invasion processes in river ecosystems is also of specific interest per se, as river ecosystems are highly diverse but also heavily affected by invasive species [19–21]. While studies focusing on invasions of larger streams are common (e.g., [7]), there are still relatively few studies that looked at invasions of smaller tributaries.

We studied amphipod community composition, the occurrence of native and non-native amphipod species, and genetic diversity and population structure (including bottleneck effects on the native species) of the most common native species in 13 spatially distinct tributary streams of Lake Constance. All 13 tributary streams drain as independent catchments into Lake Constance. The lake acts as a “mainland” habitat from which possible invasions into all tributaries can occur. Over the last decades, a series of non-native species have been introduced and rapidly spread across the whole lake [22, 23]. Subsequently, some of these non-native species may invade the tributary streams. We here focused on gammarid amphipods, of which three native species and two non-native species are known from Lake Constance [22, 24]. The three native species are naturally occurring in the lake [25] and at least two of them have also historically inhabited the tributary streams [24–26]. The two non-native species were sequentially (and accidentally) introduced into the lake itself [24–26], and had the potential to subsequently and independently invade into the tributaries. We monitored amphipod communities in linear transects along each tributary for two subsequent years. We were especially interested in the spatial occurrence of native versus non-native amphipod species, as well as the latter’s possible consequences on abundance and genetic structure of the most common native species [27].

The objective of our study was to search for possible small-scale effects of non-native amphipod species on amphipod community composition, abundance and genetic structure of native amphipod species along linear tributary streams (for large scale effects see e.g. [28, 29]). Additionally, we wanted to address possible differences in genetic population structure between outlet and headwater populations, which have been observed for aquatic invertebrates at large scales. We looked for such a patterns at smaller reach scale (tributaries of a few km), and did so across replicated tributaries, in order to study the generality of any such patterns. With invasions originating from the lake, we hypothesized (i) that community composition changes systematically along the longitudinal transect from tributary outlet at the lake to headwater sites, and expected a decrease in community richness, (ii) that invasions result in a decrease in population sizes, and (iii) that non-native species negatively affect the genetic diversity of native amphipods and caused recent bottlenecks. As a side aspect of this last hypothesis, we also tested whether headwater populations are genetically differentiated and possibly impoverished compared to outlet populations due to isolation effects.

## Methods

### Study system

Lake Constance (47°38′0″N, 9°22′0″E) is one of the largest freshwater lakes in Western Europe situated at the border triangle of Austria, Germany and Switzerland. Its mean elevation is 395 m a.s.l., with a maximum depth of 254 m. It has a total surface area of 536 km^2^, and a shore length of 273 km (72 km of which in Switzerland). The lake can be partitioned into two separate systems, the Obersee and the Untersee, connected via the Seerhein. The Obersee is mostly surrounded by agricultural land, whereas the Untersee has more forested areas alongside the shore. Lake Constance represents a well-studied system that has undergone major changes in the last century. With increasing agricultural land use, it experienced an extensive eutrophication, peaking in the 1970s. Phosphorus and other nutrient levels have subsequently been decreasing [30]. More recently, Lake Constance has been heavily affected by the arrival of a large number of non-native species, most of them invertebrates, such that community composition in the lake drastically changed within the last 30 years (e.g., [22, 23]). The river Rhine contributes the largest inflow into Lake Constance. Additionally, there are many small-scale tributaries, draining smaller, independent catchments into the lake. Each of these stream networks has a total length of only few kilometers, and the outlet and headwater sites are mostly only 1–5 km apart. These tributaries are “small but mighty” [31], because they contain a large diversity of native invertebrates and the large number of these tributaries offers a possibility to study invasion processes in a naturally replicated manner.

### Sampling design

We aimed at sampling a selection of smaller tributary streams of Lake Constance in a standardized and replicated way. We selected streams on the following criteria: (1) only smaller tributaries of two to a few km lengths were selected, (2) only short reaches (<100 m) should be built over, and (3) streams must be small enough that kicknet sampling was feasible. We preselected 21 streams fulfilling these criteria using GIS tools and openly available maps (SwissTopo, Federal Office of Topography). All streams were visited in the field, after which 8 were subsequently excluded for not fulfilling the above criteria. In the 13 remaining streams (Table 1), we established transects of sampling sites along the main stream-course. Sampling sites were located at the stream outlet (0 m site, directly at the lake), at 50, 100, 500, 1000 m and (when feasible/when the stream was large enough) at 5000 m upstream direction (Table 1). In the following, we refer to this distance as “upstream distance”. These transects were reflecting almost the whole longitudinal extent of these tributaries, and allowed comparison of outlet sites vs. headwater sites. We acknowledge that the distances of 1–5 km between outlet and headwater sites are smaller than in other studies, which, however, looked at generally much larger streams (e.g., [31]). We specifically focused on these smaller tributaries, as they contribute strongly to the overall length of streams in larger stream networks [18]. In two streams, some sites were not accessible. In the Eschlibach, the 1000 m site was on private land and inaccessible and we took a sample at 1825 m. In the Seegraben, the 500 and 1000 m sites could not be accessed due to safety issues, and one sample at 1600 m was taken instead. All sites were on publicly accessible land and no permits were required to take samples.

**Table 1:** Overview of the tributary streams sampled (ordered from West to East)

Id	Stream name	Locality	Coordinates	Location outlet	Length	Stream order
1	Speckbach	Steckborn	715,145/280,124	Untersee	4	3
2	Eschlibach	Berlingen	720,005/281,697	Untersee	3	2
3	Manebach	Salenstein	720,888/281,687	Untersee	2	2
4	Anderbach	Ermatingen	723,355/281,512	Untersee	5	4
5	Chastlerbach	Tägerwilen	727,598/280,609	Seerhein	6	4
6	Seebach	Münsterlingen	735,511/277,311	Obersee	2	2
7	Hornbach	Güttingen	739,814/275,056	Obersee	6	3
8	Romanshorner Aach	Salmsach	746,156/269,088	Obersee	17	4
9	Imbersbach	Arbon	749,316/265,670	Obersee	3	2
10	Steinach	Steinach	751,294/263,275	Obersee	>15	4
11	Hornbach	Horn	752,064/262,768	Obersee	6	3
12	Goldach	Goldach	753,549/261,830	Obersee	>15	5
13	Seegraben	Staad	758,674/261,577	Obersee	4	3

We give a unique identity number (Id), the tributary stream name, the name of the locality where it drains into Lake Constance, the coordinates of the outlet site (CH1903 coordinate system), location of the outlet, total stream length in km (measured along the longest reach), and stream order (at outlet)

### Sampling

We sampled all sites once during March 16 2012 to April 13 2012, and a second time during May 6 2013 to August 6 2013. The second sampling period started later and was prolonged due to more and stronger rainfalls in 2013, which is prohibitive and delayed sampling. Sampling sites were located in the field using a GPS device, and the sampled site had to be representative of typical conditions in that section of the stream section (we allowed a minor setoff of ±10 m to adjust for this). At each site, we conducted four kicknet samplings (one close to each of the two river banks, and the other two evenly spread across the river transect, covering the most suitable habitats) to get quantitative estimates of amphipod populations [32]. Each sample was taken by kicking 30 s in the streambed. All four samples per site were pooled, presorted to remove coarse material (leaf litter, stones) and then filtered through a sieve with 500 µm pore sizes. All organic material, including amphipods, retained in the filter was stored in 80 % molecular grade ethanol. In the laboratory, we screened all samples for amphipods, using a stereomicroscope (Leica M205C or Olympus SZH-ILLB). All amphipods were isolated and identified to the species level [33]. We excluded the smallest individuals (<3 mm) of *Gammarus fossarum, Gammarus pulex* and *Gammarus lacustris*, as at this size these three species cannot be reliably told apart morphologically.

### Genetic analyses

*Gammarus fossarum* is a known species complex, consisting of at least three species within Switzerland, and even more outside of Switzerland [34, 35]. The cryptic species can only be identified with molecular methods, and previous work [24] shows that in the Bodensee region, only *G. fossarum* type A can be found. We genotyped individuals of *G. fossarum* in order to establish its species identity. As a primary objective, we then used these genotype data to analyse if there are signals of recent bottlenecks in these populations (for analytical details see below). As a secondary objective, we also used these genetic data to measure within-species genetic diversity and genetic differentiation. In the eight streams in which we found >20 individuals of *G. fossarum* at both stream outlets and headwater reaches (stream identity number: 1, 2, 3, 4, 5, 6, 7, and 12 in Table 1), we genotyped 20–30 individuals from each reach. In one stream (Goldach) we compared the 1000 m site to the 5000 m site due to the lack of individuals at further downstream sites. DNA was extracted using the HotSHOT method (following [36, 37]). We used 10 microsatellite (gf08, gf10, gf13, gf18, gf19, gf21, gf22, gf24, gf27 and gf28) markers that were previously developed for this species [37]. PCR was conducted using multiplex amplifications [35, 37]. PCR product was diluted 1:10 in Milli-Q water (Millipore, Billerica, MA, USA) before mixing with GeneScan LIZ 500 (Applied Biosystems, Forster City, CA, USA). Samples were run on an ABI 3130xl (Applied Biosystems) and peaks were scored in the program GeneMarker^®^ Version 2.4.0 (Softgenetics, LC State Collage, PA, USA).

We tested Hardy–Weinberg equilibrium for all loci by using the web-based version of the program GENEPOP version 4.2 [38, 39] with default settings. Significance levels were adjusted using a Bonferroni correction. Locus gf08 showed heterozygote excess and thus was excluded from further analysis. We used the clustering method offered in the program STRUCTURE [40] to investigate population structure. The default parameter settings for the ancestry admix model and the correlated allele frequencies model were used as advised in the user manual of the program. Length of burn-in and Markov chain Monte Carlo were both set to 10,000. We tested cluster number (K) ranging from 1 to 15 and conducted 20 iterations for each K value. To assessed the optimal K, we used the ad hoc quantity of delta K after Evanno et al. [41].

Furthermore, we analysed allelic richness (corrected for sampling size), heterozygosity as well as M-ratio of *G. fossarum* populations from outlet vs. headwater sites, using the R package hierfstat [42]. Allelic richness and heterozygosity are established measures of the local genetic diversity, with the general expectation that they should be higher in downstream habitats. The M-ratio (the mean ratio of the number of alleles to the range in allele size), instead, is a measure for recent demographic changes (such as bottlenecks or decreases in population sizes) [43–45], with the expectation that the occurrence of non-native species may have resulted in recent bottlenecks. Most importantly, we specifically calculated the critical threshold values for recent bottlenecks (M_C_), using the Critical_M software by Garza & Williamson [45]. We calculated expected M-ratio values in the absence of recent bottlenecks as well as M_C_ (equilibrium value at which 95 % of the M values should be larger) for our studied populations sizes, using default settings for theta (10), mean size of larger mutations (3.5) and fractions of mutations that are larger than single steps (0.1). The expected critical threshold values (p < 0.05) are between 0.647 to 0.689.

### Analyses

Using linear mixed-effects models [46], we analysed the presence and abundance (square-root transformed) of amphipods using their historic origin (native versus non-native species) as well as upstream distance from the lake as fixed effect explanatory variables of interest. Stream identity, species identity and year were included as random effects. For the definition of fixed versus random effects we follow Searly et al. [47], such that effects are fixed if they are interesting in themselves and random if there is interest in the underlying population. In our case, the origin (native vs. non-native) and the upstream distance are of interest themselves. Contrarily, year, tributary identity and species are samples of larger underlying population on which the interest is.

We used likelihood ratio tests in order to attain *p* values, starting with the full model (including interactions among the two fixed effects), and sequentially comparing reduced versions of the model with each other. Additionally, we analysed community diversity (Shannon diversity index, see [48]) of all amphipod communities with upstream distance as fixed effect, and year and stream identity as random effects.

Finally, we compared the genetic diversity of *G. fossarum* populations (comparing allelic richness, heterozygosity and the M-ratio between stream outlets and headwater reaches) using linear mixed effects model [46]. We used rarefied allelic richness [42] as response variable of interest. Position within the stream (outlet vs. headwater) was taken as fixed effect, while stream identity and locus (using the nine different microsatellite loci) were added as random effect. When not mentioned differently, all analyses were done in R [49] using the respective packages given.

## Results

In total, we found amphipods belonging to five different species. 4839 individuals belonged to *Gammarus fossarum,* 2148 individuals belonged to *Gammarus roeseli,* 391 individuals belonged to *Dikerogammarus villosus,* 56 individuals belonged to *Gammarus pulex,* and 20 individuals belonged to *Gammarus lacustris* (see Additional file 1) .

**Fig. 1:**
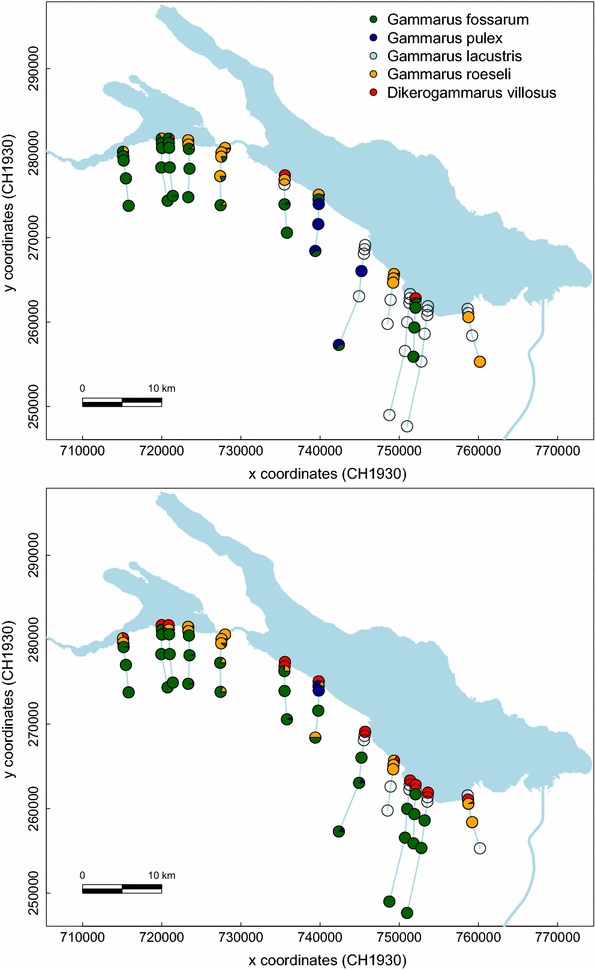
Community composition and diversity of amphipods in 13 tributary streams of Lake Constance (*light blue*) in 2012 (**a**) and 2013 (**b**). Local amphipod community composition is given with pie-charts for each site sampled, starting at the stream outlet (lake site) towards increasing upstream distance (0, 50, 100, 500, 1000 and 5000 m sites respectively, arranged from North to South). Pie-charts give the relative proportion of amphipod species for each site. *Gammarus roeseli* and *Dikerogammarus villosus* are non-native species, while the three other species are natives. For clarity, position and river-line of the 13 tributary streams are slightly schematised and shifted

We found a highly significant interaction between the occurrence of native versus non-native amphipods and upstream distance (*Chi*^2^ value = 16.03, *p* value <0.0001; Fig. 1), reflecting that non-native species were mostly present at the outlet, while the native species were mostly present at the further upstream sites. The variance in presence of amphipods was mostly explained by this interaction term, and subsequently the main effects of origin of amphipods (native vs. non-natives) and upstream distance were individually not significant (for distance: *Chi*^2^ value = 0.297, *p* value = 0.59; for origin: *Chi*^2^ value = 0.006, *p* value 0.94).

**Fig. 2:**
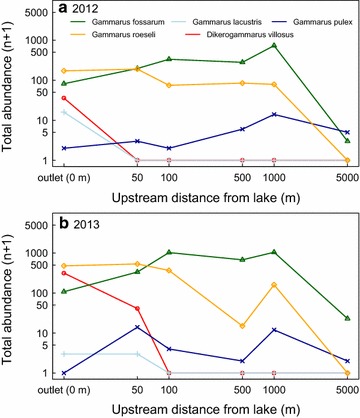
Abundance of amphipod species in 13 tributary streams of Lake Constance in 2012 (**a**) and 2013 (**b**). Abundance (log[n + 1]) is given for each species relative to the sampling sites’ upstream distance from lake (stream outlet). *Gammarus roeseli* and *Dikerogammarus villosus* are non-native species, while the three other species are natives

In an analogous finding, there was a significant interaction between the abundance of native versus non-native amphipods and upstream distance (*Chi*^2^ value = 5.62, *p* value = 0.018; Fig. 2), reflecting that non-native species were more common at the outlet, while the native species were more common at the upstream headwater sites. The variance in abundance of amphipods was mostly explained by this interaction term, and subsequently the main effects of origin of amphipods (native vs. non-natives) and upstream distance were individually not significant (for distance: *Chi*^2^ value = 5.78, *p* value = 0.06; for origin: *Chi*^2^ value = 5.57, *p* value 0.12). When analyzing community diversity (Shannon-index) we did not find a significant effect of upstream distance on diversity (*Chi*^2^ value = 0.05, *p* value = 0.82).

Abundance of *G. fossarum* was significantly higher at sites without *D. villosus* compared to sites with *D. villosus* (Wilcoxon signed rank test, *W* = 1550.5, *p* = 0.019). Abundance of *G. fossarum* did not differ significantly between sites with or without *G. roeseli* (Wilcoxon signed rank test, *W* = 1995, *p* = 0.99).

### Genetic results

A total of 478 individuals of *G. fossarum* from eight streams were used for the microsatellite analysis (see Additional file 2). Based on the genotype data, we identified all individuals to be part of the *G. fossarum* clade A [35, 37, 50]. We found highest probability for a cluster of K = 6 by using Evanno-Correction [41]. Populations mostly cluster within streams with some streams clustering together (Eschlibach and Manebach, and Seebach and Goldach) when assuming K = 6.

Rarefied allelic richness (rarefied to 32 allelic counts [42]) varied across sites and loci. Rarefied allelic richness across loci ranged from 2.08 to 8.50 alleles per locus. Mean allelic richness across all loci ranged from 4.69 to 6.53 alleles per site. There was no significant effect of position within the stream on allelic richness (mean ± se rarefied allelic richness was 5.21 ± 0.31 at headwater versus 5.29 ± 0.33 at outlet sites*; Chi*^2^ value = 0.05, *p* value = 0.82; Fig. 5a). Furthermore, there was no significant effect of position within the stream on heterozygosity (mean ± se heterozygosity was 0.54 ± 0.073 at headwater versus 0.55 ± 0.074 at outlet sites, *Chi*^2^ value = 0.04, *p* value = 0.95; Fig. 5b) nor on the M-ratio (mean ± se M-ratio was 0.40 ± 0.013 at headwater vs. 0.42 ± 0.013 at outlet sites, *Chi*^2^ value = 0.06, *p* value = 0.8; Fig. 5c).

Importantly, however, in all populations we found significantly lower M-ratio values than expected under equilibrium. Expected critical equilibrium values (M_C_, [45]) for our sample sizes are between 0.647 to 0.689, while all observed values were below 0.5 (Fig. 5c). This is a significant (p < 0.05) indication for recent bottlenecks in all of these populations.

## Discussion

Non-native species and their spatial distribution have been studied for many decades (e.g., [51, 52]). However, most of this research has been limited by the lack of replicated realizations of the invasion process. In riverine systems, much of the observed invasion patterns are unique incidences (e.g., [7]), hindering generalizations, and the focus has been on larger rivers. Here, we studied community composition and the occurrence of non-native amphipods as well as genetic population structure of the most common native amphipod species in naturally replicated, smaller tributary streams of Lake Constance in Central Europe (Fig. 1).

**Fig. 3:**
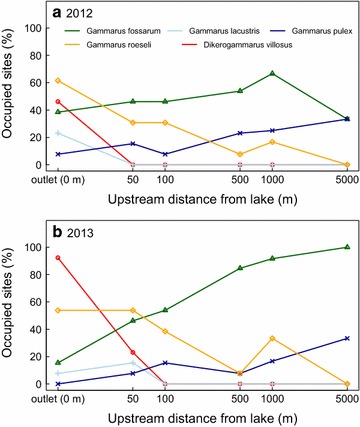
Percentage of sites occupied by each of the five amphipod species across the 13 tributary streams of Lake Constance in 2012 (**a**) and 2013 (**b**). Percentage of sites occupied is given for each species relative to the sampling sites’ upstream distance from lake (stream outlet). *Gammarus roeseli* and *Dikerogammarus villosus* are non-native species, while the three other species are natives

We found spatially stable patterns of community composition and occurrence of non-native gammarid amphipod species along 13 independent tributary streams of Lake Constance. Specifically, occurrence as well as abundance of two non-native amphipod species decreased from the stream outlets at the lake site towards upstream headwater reaches (Figs. 2, 3). The non-native amphipod species arrived in the Lake and subsequently colonized tributary streams [25, 26]. One of the non-native species (*G. roeseli*) was introduced in the system around 1850 and is well-established in the lake [24], while the other (*D. villosus*) is a much more recent invader [16], and was first observed in the lake in 2002/2003 [24, 26]. Both species have completely colonized the lake shores around Lake Constance, and, especially well-documented for *D. villosus*, can be found all around the lake from 2007 onwards [26]. The spatio-temporal patterns of this invasion across the lake is documented on http://www.neozoen-bodensee.de/neozoen/dikerogammarus. Interesting, however, the potential to colonise all tributaries equally, at least from a spatial perspective, was not realized: only some tributaries got colonized to greater or lesser degree by either of the species, indicating that there are some inherent stochastic aspects involved. Across all colonized tributaries, the more historic invader *G. roeseli* reached much further upstream (including our most upstream sampling sites) compared to the very recent invader *D. villosus*, which was mostly confined to the stream outlets. Our study did not find systematic differences across the 2 years, and it will be up to future research to see if the current invasion fronts are stabilizing or still propagating. Populations of the native *G. fossarum* were largest at the uppermost headwater reaches, and showed some genetic differentiation between the different tributaries. Contrary to our expectation, however, no longitudinal genetic differentiation within individual tributaries was found. This suggests no limits to gene flow and dispersal within tributaries.

It has been argued that non-native species from the Ponto-Caspian region mostly originate from the potamal sections of large rivers and thus are also confined to these habitats in the invaded region [53]. This could explain the predominant occurrence of *D. villosus* at stream outlets and lakeshores [54]. However, the downstream sites of four of the tributary streams were medium sized (e.g., stream identity numbers 5, 8, 10, and 12; see Table 1), and had modified riverbanks, consisting of large boulders, which are favoured habitat of *D. villosus* [55]. The species’ absence from these sites can thus not be explained by habitat quality only, as the species’ ecological requirements are fulfilled in at least some of the further upstream headwater reaches [56]; this is also indicated by the species’ presence in other riverine systems [24, 57]. We hypothesize that the observed spatial patterns of non-native species could reflect an ongoing invasion front, and that invaders might still be moving upstream, just in small numbers. Our kicknet method was a site-specific sampling method commonly applied to monitor aquatic benthic organisms and the method is unable to reliably detect organisms at low to very low densities (e.g., as commonly found at invasion fronts) and isolated populations in between sampling locations. Thus, we cannot rule out the occurrence of individuals or even small populations of the non-native species at further upstream sites (e.g., detectable through environmental DNA technologies, see [58, 59]). These occurrences would, however, be small and arguably ecologically less relevant.

We then specifically looked at two aspects of the genetic structure of the most common native species (*G. fossarum*), and analysed firstly within and between genetic diversity secondly potential recent bottlenecks arising from the invasion. These two aspects are now sequentially discussed.

**Fig. 4:**
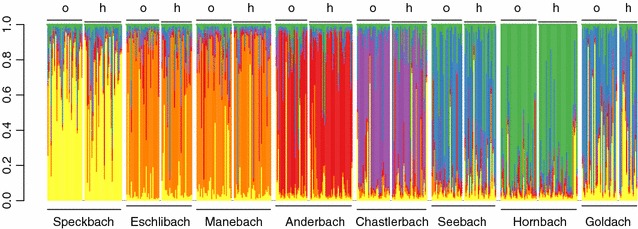
Estimated genetic population structure of *Gammarus fossarum* in eight tributary streams of Lake Constance, based on microsatellite data. The *graph* is based on the STRUCTURE run of the highest estimated probability run with K = 6. Each individual is represented with a single *vertical line* partitioned into *different colours*. *Each colour* represents a different cluster. Streams are ordered in the figure from west to east. Within stream, individuals either originated from the stream outlet (*o*) or the headwater reaches (*h*)

**Fig. 5:**
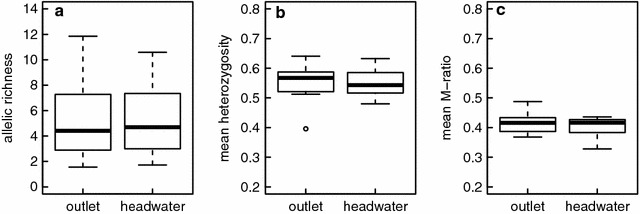
**a** Allelic richness of *Gammarus fossarum* in headwater and outlet populations. **b** Heterozygosity of *G. fossarum* in headwater and outlet populations. **c** M-ratio of *G. fossarum* in headwater and outlet populations. *Boxplots* show the median (*solid line*) and 25 and 75th percentiles (*boxes*) and values ≤ 1.5 times the range (*whiskers*). Values larger than that are given *as points*

We observed some genetic differentiation of *G. fossarum* between streams (Fig. 4), suggesting that dispersal and gene flow between tributaries is reduced, and that they are relatively isolated and independently. However, this isolation was far from complete, and in some streams (e.g., Goldach, Seebach) there were strong signals of gene flow. Allelic richness and heterozygosity were not different between headwater and outlet populations (Fig. 5). This is contrary to various studies that predicted and found a decrease in genetic diversity and heterozygosity in headwater to outlet sites [31, 43, 60]. We see several mutually non-exclusive explanations for our finding. First, it is possible that these patterns may depend on the scale looked at: we looked at spatial dimensions of about one order of magnitude smaller than past work (e.g., [7, 14]). At this scale the proposed effects may be no longer as relevant and it is also more difficult to detect any difference. Second, it has been shown that the decrease in genetic structure towards headwater sites may not be as universal in rivers systems as previously thought [61], and that headwater populations may be as diverse and act as reservoir populations.

When looking at the M-ratio and critical M values, we found in all populations significant signals of recent bottlenecks [51]. Interestingly, there was no difference between headwater and outlet populations with respect to the bottleneck signal. Given that our data are correlative, we cannot give a causal explanation for these indications of recent bottlenecks. Possible, mutually non-exclusive explanations are recent bottlenecks due to environmental disturbances (especially pollution) known from that area between the 1950s to the 1980s, partial desiccation of the streams and subsequent population bottlenecks or the arrival of non-native species. Given that the M-ratio has been shown to be a good indicator of recent demographic changes [44], the absence of a difference in the bottleneck signal between headwater versus outlet populations suggests that demographic processes were not different along the longitudinal transects in these stream populations. This likely precludes non-native species as the sole driver of the bottlenecks, because there was no evidence of non-native species at headwater populations, while these headwater populations still showed the bottleneck signal.

Based on theoretical concepts on asymmetric dispersal and network structure [60], it has been proposed and experimentally shown that both species and genetic diversity in dendritic riverine ecosystems decreases with increasing upstream position (e.g., [31, 44, 62, 63]) (but see [61]). Overall, we see two mutually non-exclusive explanations for not finding an effect of upstream position on neither species nor genetic diversity. First, it is possible that the distances surveyed are too small to reveal any signal of upstream position on species or genetic diversity. Second, it is possible that a historically higher diversity at stream outlet sites has been decreased through the predominant occurrence of non-native species at these sites, thereby levelling out any previously existing diversity gradient. Indeed, we found a pronounced shift in community composition from non-native dominated communities at outlet sites to native dominated communities at headwater sites. For *G. lacustris* it is known that occurrence in the lake declined drastically after the arrival of *D. villosus* [23].

## Conclusions

In conclusion, we find that invasions of two non-native amphipod species into naturally replicated tributary streams result in distinct spatial community patterns of their occurrence predominantly at the outlet sites. On contrary, the most upstream headwater sites were refugia for the most common native amphipod species. While the non-native species had significant effects on the occurrence and abundance of native species, we surprisingly did not find a distinct longitudinal signal in the genetic structure of the most common native amphipod species. This suggests, in analogy to other metapopulations of crustaceans [64], an important role of small and isolated populations, and an absence or lag of imprinting effects of non-natives species on the population genetic structure of native species.

## Additional files

10.1186/s12898-016-0079-7 The complete dataset on the community data, including a unique identification number for each stream (“nr”), the stream name (“stream_name”), the locality (“gemeinde”, political community), the coordinates (“x_koord” and “y_koord”) in the Swiss coordinate system, the upstream distance to the lake (“distance”, in m), the site being at Untersee or Obersee of Lake Constance (“lakepart”), the sampling day, month and year, the amphipod species identity (“species”) and the number of individuals of that species found (“individuals”).
10.1186/s12898-016-0079-7 The complete microsatellite dataset, in which we give for each *Gammarus fossarum* genotyped a unique identification number (“sort_nr”), the position of the respective population (“position”; headwater h or outlet o), the population number (“pop”), a unique genotype number (“Individuum”), the stream name (“Bach”), and then all the microsatellite data values for the 10 microsatellite loci gf13, gf18, gf22, gf27, gf10, gf21, gf24, gf19 and gf28.
